# Comprehensive analysis of peripheral blood non-coding RNAs identifies a diagnostic panel for fungal infection after transplantation

**DOI:** 10.1080/21655979.2022.2032963

**Published:** 2022-02-06

**Authors:** Anli Yang, Huadi Chen, Jianwei Lin, Ming Han, Xiaopeng Yuan, Tao Zhang, Qingwei Nian, Mengran Peng, Dian Li, Chenglin Wu, Xiaoshun He

**Affiliations:** a Organ Transplant Center, The First Affiliated Hospital, Sun Yat-sen University, Guangzhou, China; b Department of Breast Oncology, Sun Yat-sen University Cancer Center, State Key Laboratory of Oncology in South China, Collaborative Innovation Center for Cancer Medicine, Guangzhou, China; c Guangdong Provincial Key Laboratory of Organ Donation and Transplant Immunology, Guangzhou, China; d Guangdong Provincial International Cooperation Base of Science and Technology (Organ Transplantation), Guangzhou, China; e Department of Hepatobiliary and Pancrease Surgery, Shenzhen People’s Hospital, Shenzhen, China; f Dermatology Department, The Third Affiliated Hospital of Sun Yat-sen University, Guangzhou, China; g Department of Data Science, Dana Farber Cancer Institute, Harvard School of Public Health, Boston, Massachusetts, USA

**Keywords:** Fungal infection, diagnosis, transplantation, non-coding RNAs

## Abstract

The occurrence of fungal infection seriously affects the survival and life quality of transplanted patients. The accurate diagnosis is of particular importance in the early stage of infection. To develop a novel diagnostic method for this kind of patient, we established a post-transplant immunosuppressed mice model with fungus inoculation and collected their peripheral blood at specific time points after infection. After screening by microarray, differentially expressed miRNAs and lncRNAs were selected and homologously analyzed with those of human beings from the gene database. These miRNAs and lncRNAs candidates were validated by qRT-PCR in peripheral blood samples from transplanted patients. We found that, compared with normal transplanted patients, the levels of miR-215 and miR-let-7 c were up-regulated in the plasma of patients with fungal infection (*P* < 0.01), while levels of miR-154, miR-193a, NR_027669.1, and NR_036506.1 were down-regulated in their peripheral blood mononuclear cells (*P* < 0.01). Principal component analysis shows that the expression pattern of the above RNAs was different between the two groups. A 6-noncoding-RNA detection panel was established by the support vector machine analysis, whose area under the ROC curve was 0.927. The accuracy, precision, sensitivity, and specificity of this model were 0.928, 0.919, 0.944, and 0.910, respectively. Though our detection panel has excellent diagnostic efficacy, its clinical application value still needs to be further confirmed by multi-center prospective clinical trials.

## Graphical Abstract

**Figure:**
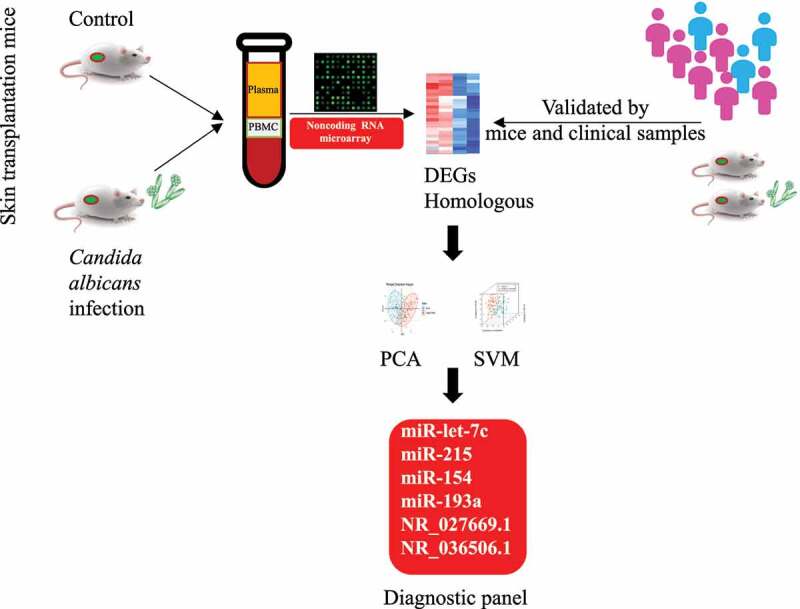

## Introduction

With the development of surgical techniques and organ preservation technology of the late 20th century and the application of novel immunosuppressants, the prognosis of organ transplant recipients has improved significantly [1]. Nowadays, infections, rejections, and cardiovascular and cerebrovascular diseases have replaced surgical complications as the three main factors restricting the survival of post-transplant patients [2–4]. Since transplanted patients are in a state of immunosuppression, they are susceptible to various infections, and infections in such patients are inclined to spread and deteriorate rapidly [5]. What’s worse, their different infection spectrum and atypical clinical manifestations increase the difficulty in early diagnosis. Therefore, to further improve their long-term survival and quality of life, more attention should be paid to the issue of infection.

Particularly, fungal infection poses a huge threat to the survival of patients and grafts [6,7]. According to reports, although the incidence of fungal infection after transplantation is approximately 13.5%, the subsequent invasive infection has a higher mortality rate than common bacterial and virus infections, which is around 70.8% [8,9]. As the most frequent pathogenic fungus, *Candida albicans* (67.5%) infections manifest as pneumonia, peritonitis, empyema, candidemia, etc. Moreover, the incidence of aspergillosis, cryptococcosis, and mucormycosis has been rising in recent years. Changes in the spectrum of infectious fungi increase the difficulty and pressure of early clinical diagnosis. Additionally, in the case of insufficient diagnostic evidence, the excessive use of anti-infective drugs increases not only the treatment cost and economic burden of patients but also the risk of adverse reactions. Hence, a novel method for the early diagnosis of fungal infection in posttransplant patients is in urgent need.

MicroRNAs (miRNAs), containing 20–25 nucleotides, are a type of endogenous non-coding RNAs (ncRNAs) with regulatory function [10]. They participate in the various biological processes including cell cycle, metabolism, organogenesis, infection defense, etc. And their expressions are highly conversed, time-ordered and tissue-specific, which can reflect the dynamic changes in the body quickly [11–13]. Long non-coding RNAs (lncRNAs), consisting of more than 200 nucleotides, are a class of ncRNAs regulating gene expression at epigenetic, transcriptional, and post-transcriptional [14]. They take part in various important life activities and are closely related to the generation, development, and progression of human diseases. Studies have disclosed their roles in cancers, hematopoiesis, and immunity [15–18]. In addition, the simultaneous detection of ncRNAs in plasma and peripheral blood mononuclear cells (PBMCs) can more fully reflect the dynamic changes of the internal environment, including those released by the infection site and immune cells. However, the diagnostic value of miRNA and lncRNA in patients’ fungal infections remains unknown, especially in that post-transplantation.

We believe that dynamically changing ncRNAs can sensitively reflect alterations in the body’s state. Therefore, we are going to use this feature of ncRNAs to establish an accurate diagnostic method of fungus infection for transplanted patients in the early stage. Here, we discovered the differentially expressed profiles of miRNA and lncRNA in the peripheral blood of normal and fungus-infected patients after organ transplantation. Furthermore, based on quantitative reverse transcription-polymerase chain reaction (qRT-PCR) verification and clustering methods, we found new biomarkers and established a diagnostic panel for detecting fungal infection in transplanted recipients.

## Methods

### Study design

**Figure 1.**
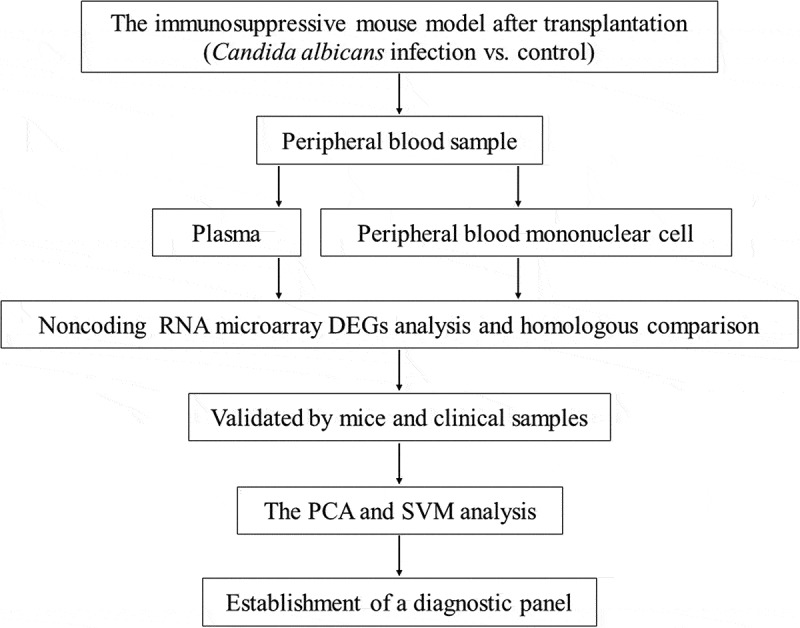
Flow chart of the study design.

Our study was conducted as follows (Figure 1). Firstly, we set up a mouse model of fungal infection after skin transplantation and collected its peripheral blood sample. RNA was extracted from plasma and peripheral blood mononuclear cells respectively. We identified different expressed miRNAs and lncRNAs by microarrays and further performed homology comparisons. Moreover, the candidate miRNAs and lncRNAs were verified in specimens from the mouse model and clinical patients. Finally, based on the expression of 6 selected non-coding RNAs, we built up a diagnostic panel of fungus infection after transplantation by clustering methods and further tested its efficiency.

### Animal model construction

The male C57BL/6 J mice (6–8 weeks old) were used as donors and Balb/c mice were used as recipients. Then we performed skin grafts on their lower back, and we used Cyclosporin A (CsA; Novartis Pharma Ltd; Germany; 30 mg/kg/day) for the immunosuppressive treatment [14]. On the 7th day after skin grafting, 200ul of 5 × 10^5^ CFU/ml *candida albicans* (ATCC®MYA-2876™) suspension was dripped into the mouse nostrils to obtain a candidiasis model. The peripheral blood was collected 3 days after infection and that of the control group was collected at the same time.

### Peripheral blood isolation and preservation

We collected the peripheral blood of mice and patients, processed with EDTA anticoagulant, centrifuged at 200 g and 4°C for 15 minutes, sucked out the upper plasma, and stored in the refrigerator at −80°C. The lower cells were resuspended in the same volume of PBS, transferred to another centrifuge tube with the same volume of peripheral lymphocyte separation medium (GE, USA, 17,144,003), centrifuged at 450 g and 4°C for 20 minutes, and then transferred the milky white lymphocyte cells at the second layer to another centrifuge tube. And then we added 10 ml PBS buffer for cell wash and repeated the above centrifuge steps, discarded the upper layer of liquid, and added 1 ml TRIzol medium (ThermoFisher, USA, 15,596,026) to dissolve the lower layer of the precipitate. Lastly, the samples were stored in the refrigerator at −80°C after pipetting repeatedly.

### RNA extraction and reverse-transcription PCR

According to the manufacturer’s instructions (ThermoFisher, USA, 15,596,026), total RNA was isolated by TRIzol reagent. And 1 μg of total RNA was subjected to reverse transcription (RT) in 20 µl reaction volumes. For the miRNA reverse-transcribed, All-in-One™ miRNA First-Strand cDNA Synthesis Kit (Genecopoeia, USA, QP007) was adopted. In the lncRNA RT reaction, we used PrimeScriptTM RT Master Mix (TAKARA, Japan, RR036A). During the Real-time PCR experiment, Power SYBR PCR Master Mix (Genecopoeia, USA, 4,367,659) was adopted and 2 µL of the cDNA was used as a template. An ABI PRISM 7500 Real-time PCR System (ThermoFisher, USA) was used to conduct amplification reactions, which run with 45 thermocycles of 30s at 94°C, 30s at 55°C, and 30s at 72°C. The primer sequences were listed in **Table S1** and **S2**. Expression levels of each tested gene were determined by the 2^−ΔΔCt^ method. U6 was used as the internal control for miRNAs, while GAPDH was used as the internal control for lncRNAs.

### Gene chip detection

For miRNA, miRCURY™ Hy3™/Hy5™ fluorescent labeling kit (Exiqon, Denmark, 208,031-A) was used to label RNA following the instructions. Then 25ul of labeled samples and 25ul of hybridization buffer were mixed, denatured at 95°C for 2 minutes and incubated on ice for 2 minutes, and transferred to a miRCURYTM LNA Array chip (Exiqon, Denmark, v.18.0). It was further processed in 12-Bay Hybridization System (Hybridization System-Nimblegen Systems, Inc., USA) at 56°C for 16–20 hours. After several rinses and drying, it was detected by Axon GenePix 4000B chip scanner (Molecular Devices, USA) for scanning and collecting relevant signal strength. Only when the strength of each plate was greater than 30, could the sample signals be used for standardized calculation and statistics.

For lncRNA, we purified the samples by mRNA-ONLY™ Eukaryotic mRNA Isolation Kit (Epicenter, USA, MOE51010), amplified, and transcribed into fluorescent cDNA. 1ul of the cDNA is mixed with 5ul 10X of blocking reagent and 1ul 25X of separation buffer at 60°C for 30 minutes, and then be diluted by 25ul 2X of GE hybridization solution. It was further transferred into Mouse LncRNA Array (Arraystar, USA, v2.0) and incubated in an Agilent hybridization oven at 65°C for 17 hours. Agilent DNA microarray scanner (Agilent, USA, G2505C) was adopted for collecting data, and Agilent Feature Extraction software (Agilent, USA, 11.0.1.1) and GeneSpring GX v12.0 software (Agilent, USA, v12.0) were used for standardization and calculation [19].

### Clinical samples collection

A total of 1008 specimens of patients with fever after organ transplant were collected from June 2014 to December 2015 in the First Affiliated Hospital of Sun Yat-sen University, whose infection spectrum covers common bacteria, viruses, and fungi (*Candida, Aspergillus, Rhizopus*, and *Cryptococcus*). Patients after liver transplant, kidney transplant, simultaneous kidney-pancreas transplant, simultaneous liver-kidney transplant, or upper abdominal multiple organ transplant were included. After fungi detection, 67 patients were diagnosed with fungus infection, which was further analyzed. And 72 peripheral blood samples of uninfected patients in the early and long-term after organ transplantation were collected as normal controls. The inclusion criteria for patients with fever are as follows [1]: clinically confirmed or highly suspected infection [2]; body temperature is higher than 38.5°C [3]; without anti-infection treatment or fever repeatedly even after anti-infection treatment. And the exclusion criteria are as follows [1]: fever caused by acute rejection or drug allergies [2]; severe coagulation disorders, blood system diseases, or in critical condition [3]; have any signs of tumor occurrence or recurrence. The study protocol was approved by the Ethical Committee of The First Affiliated Hospital of Sun Yat-sen University (No. 2,013,102) and was conducted according to the Declaration of Helsinki principles. Informed consent was obtained from all participants.

### Fungi detection

Identification of pathogens was carried out in specimens of fever patients. Specimens for fungi detection were obtained according to the patient’s clinical symptoms and the judgment of the doctor in charge, including peripheral blood, sputum, abdominal drainage fluid, wound exudate, etc. If the detection result of sputum is positive, it indicates the patient has a lung infection. A similar analogy for other results (Figure S1). *Candida* was detected by fungus culture and further verified by VITEK 2 Yeast Identification Card (YST) with the help of VITEK 2 Compact (bioMerieux, France). Besides, we detected *Rhizopus* by fungus culture, which was further confirmed by mass spectrometry. We adopted colloidal gold immunochromatography (Immuno-Mycologics, Inc., USA) to detect *Cryptococcal* antigens, which was confirmed again by ink staining. Additionally, *Aspergillus* antigens and antibodies were detected with a diagnostic kit (Bio-Rad Laboratories, China) using an automatic enzyme-linked immunoassay analyzer (CRED Medical Equipment Co., Ltd., China). When the result was positive, such a sample would be selected for miRNA or lncRNA detection. The demographic and clinical features of these patients are shown in **Table S3**.

### Statistics and analysis

We pick out differently expressed miRNA and lncRNA by Volcano Plot and adopt MEV for cluster analysis. miRbase and blast are used for the humanization comparison of miRNA and lncRNA. SPSS19.0 software is adopted for statistical analysis and the experimental data are presented as mean ± standard deviation. All hypothesis tests are two-sided and a *P* value of <0.05 was determined as statistically significant. Pearson correlation analysis is used for identifying the correlation between each index and the Principal Component Analysis (PCA) is adopted for recognizing the effective index. Then we establish a mathematical model for the diagnosis of infection with the help of Supported Vector Machine Analysis (SVM). The e1071 (Probability Theory Group, Wien), caret (Max Kuhn), and pROC packages in R version 3.6.3 (R Core Team, Austria) are used to train a binary supporting vector machine (SVM) classifier with C-classification SVM-Type and linear SVM-kernel [20].

## Results

To take advantage of the dynamically changing ncRNAs, which sensitively reflect alterations in the body’s state, to establish an accurate diagnostic method of fungus infection for transplanted patients in the early stage, a model of fungal infection after skin transplantation in mice was constructed. We collected peripheral blood samples and extracted RNA from plasma and peripheral blood mononuclear cells (PBMC) for microarray screening.

### Discover differentially expressed miRNA and lncRNA

**Figure 2.**
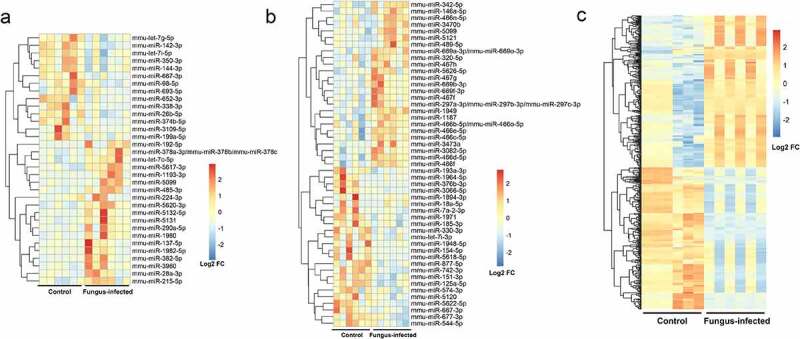
Discover differentially expressed miRNA and lncRNA in fungus-infected mice after transplant. (a) Heatmap of differentially expressed miRNAs in the plasma. (b) Heatmap of differentially expressed miRNAs in the peripheral blood mononuclear cell. (c) Heatmap of differentially expressed lncRNAs in the peripheral blood mononuclear cell.

Detected by gene chip, we have found out 19 up-regulated miRNAs and 14 down-regulated miRNAs in plasma as well as 25 up-regulated miRNAs, 24 down-regulated miRNAs, 252 up-regulated lncRNAs, and 236 down-regulated lncRNAs in PBMC of the fungus-infected mice when compared with the control ones (Figure 2a, B, C).

After humanization comparison by miRbase and blast, we have found that when compared with the control group, there were 16 up-regulated miRNAs and 11 down-regulated miRNAs in plasma as well as 3 up-regulated miRNAs, 11 down-regulated miRNAs, 33 up-regulated lncRNAs, and 24 down-regulated lncRNAs in PBMC of the fungus-infected group.

### Novel biomarkers for fungal infection after organ transplant

**Figure 3.**
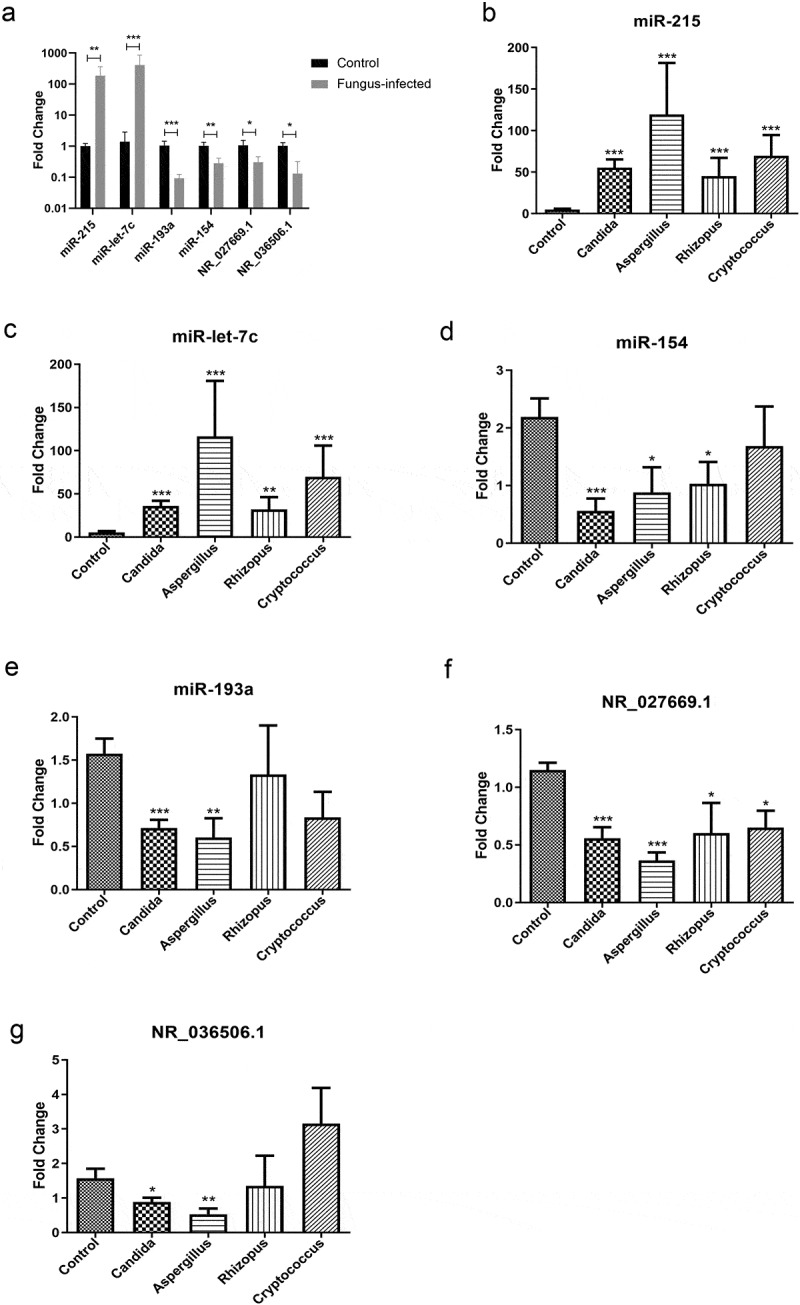
Identification of novel biomarkers for fungal infection after organ transplantation. (a) Real-time quantitative PCR validation of differentially expressed miRNAs and lncRNAs in the peripheral blood sample from a murine model of fungal infection. (b) Verification of miR-215 in plasma from transplanted patients with various fungal infections. (c) Validation of miR-let-7 c in plasma from transplanted patients with different kinds of fungus infection. (d) Expression of miR-154 in PBMC from transplanted patients with various fungal infections. (e) Validation of miR-193a in PBMC from transplanted patients with different kinds of fungus infection. (f) Verification of NR_027669.1 in PBMC from different fungus-infected patients after transplantation. (g) Expression of NR_036506.1 in PBMC from posttransplant patients with various fungal infections.

After RT-PCR verification in mouse peripheral blood samples, we pick out 4 miRNAs (2 in plasma and 2 in PBMC) as well as 2 lncRNAs from the differentially expressed genes (Figure 3a). Through further validation in the peripheral blood samples from patients, we found that the levels of miR-215 and miR-let-7 c were up-regulated in patients with fungal infection when compared with normal patients after transplant (*P* < 0.01) (Figure 3b, C). While levels of miR-154, miR-193a, NR_027669.1, and NR_036506.1 were down-regulated in their PBMC (*P* < 0.01) (Figure 3c, D, E, F).

Compared with those healthy recipients in the control group, the expression of miR-215 rose to 11.3 times in the plasma of patients with *Candida* infection, 24.2 times in patients with *Aspergillus* infection, 9.2 times in patients with *Rhizopus* infection, and 14.2 times in patients with *Cryptococcus* infection (Figure 3b). The average increased level was 13.5-fold in the fungus-infected group.

At the same time, the average increased level of miR-let-7 c in the plasma of the fungal infection group was 9.8 times when compared with the control one. To be specific, its level increased 6.8 times in patients with *Candida* infection, 22.0 times in patients with *Aspergillus* infection, 6.1 times in patients with *Rhizopus* infection, and 13.2 times in patients with *Cryptococcus* infection (Figure 3c).

On the contrary, in the PBMC of all patients with fungal infections, miR-154 dropped to an average of 34.9% of the control group. Describe in detail, its expression level fell to 25.7% in patients with *Candida* infection, 40.2% in patients with *Aspergillus* infection, and 47.1% in patients with *Rhizopus* infection. But there were no significant changes in patients with *Cryptococcus* infection (Figure 3d).

On the other hand, the level of miR-193a in the PBMC of infection group fell to an average of 49.1% in that of the control one. Specifically, its expression level dropped to 45.4% in patients with *Candida* infection and 38.4% in those with *Aspergillus* infection. However, there are no significant changes in patients with *Rhizopus* or *Cryptococcus* infection (Figure 3e).

Similarly, in the PBMC of all patients with fungal infections, the level of NR_027669.1 dropped to an average of 46.8% of the control group. To be precise, it fell to 48.4% in patients with *Candida* infection, 31.6% in those with *Aspergillus* infection, 52.2% in patients with *Rhizopus* infection, and 56.5% in patients with *Cryptococcus* infection (figure 3f).

Compared with those healthy patients after transplant in the control group, NR_036506.1 level in PBMC fell to 56.6% in patients with *Candida* infection and 33.3% in those with *Aspergillus* infection. But there are no significant changes in patients with *Rhizopus* or *Cryptococcus* infection. Though no significant differences between the whole infection group and the control one, the expression level in the infection group (excluding patients with cryptococcosis) dropped to an average of 55.8% of the control one (Figure 3g).

### The expression of different biomarkers in patients with various infection sites

We further analyzed the expression of various biomarkers in patients with different infection sites, such as lung, blood, abdominal cavity, and surgery incision (Table 1). Compared with normal patients, miR-215 and miR-let-7 c in patients with infections in the above 4 sites were up-regulated significantly, while the level of NR_027669.1 was down-regulated significantly. Moreover, in patients with lung or blood infection, the levels of miR-154 and miR-193a were down-regulated notably, but there were no significant changes in patients with incision infection. Besides, the level of miR-193a in patients with infection in the abdominal cavity was down-regulated markedly. But the level of NR_036506.1 was down-regulated strikingly only in patients with infections in the lung or abdominal cavity.

**Table 1.** The expression of different biomarkers in patients with various fungal infection sites

Infection site	Lung	Blood	Abdominal cavity	Surgery incision
miR-215	<0.001	<0.001	<0.001	<0.001
miR-let-7 c	<0.001	<0.001	<0.001	<0.01
miR-154	<0.001	<0.05	0.08	0.25
miR-193a	<0.001	<0.01	<0.001	0.86
NR_027669.1	<0.001	<0.001	<0.05	<0.05
NR_036506.1	<0.05	0.25	<0.05	0.15

Comparisons between normal transplanted patients and patients with fungal infections in different sites were conducted, and the *P* value of each comparison was shown.

### Expression pattern analysis of biomarkers and establishment of classification model for the diagnosis of fungal infection after transplantation

**Figure 4.**
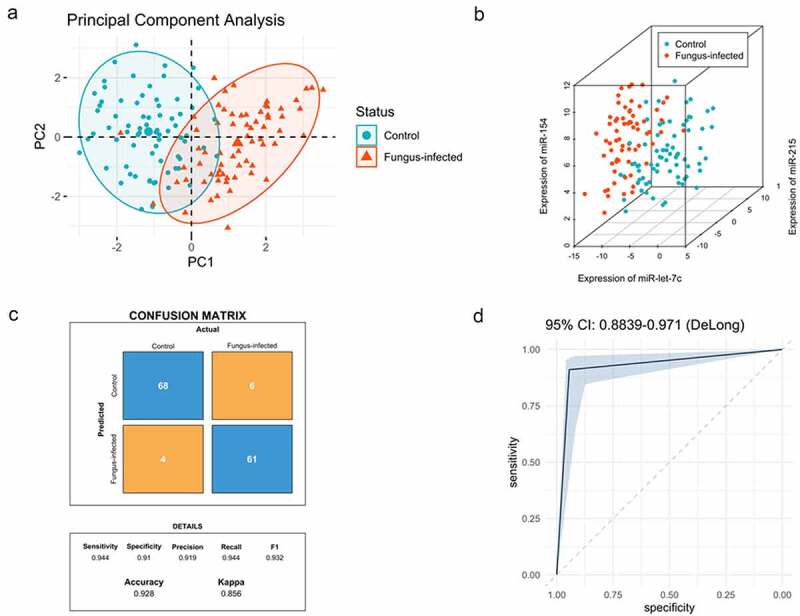
Expression pattern analysis of biomarkers from normal and fungal infected transplant recipients and classification model for normal and fungal infection (a) PCA analysis between normal and fungal infected patients after organ transplantation. (b) SVM analysis between normal and fungal infected patients after organ transplantation. (c) The confusion matrix method was used to test the model performance, and its accuracy, precision, sensitivity, and specificity were shown below. (d) The receiver operating characteristic (ROC) analysis was conducted with 2000 stratified bootstrap replicates.

PCA analysis showed that the expression pattern of miRNAs and lncRNAs was different between normal patients and patients with fungus infection after transplantation (Figure 4a). The PC1 and PC2 explained 62.7% of the total variance (**Table S4**).

Data of biomarkers (dCT) from both groups were analyzed in RStudio (RStudio Team, USA), including 72 cases in the control group and 67 cases in the fungus-infected group (Figure 4b). The fitted equation was as follows: Y = Beta1 * miR-let-7 c + Beta2 * miR-215 + Beta3 * miR-154 + Beta4 * miR-193a + Beta5 * NR_027669.1 + Beta6 * NR_036506.1 + Bias, with Beta = [0.970; 0.846; −0.768; −0.219; −0.818; −0.229], Bias = 0.249.

We have located a total of 34 support vectors. We first tested the model performance using the confusion matrix method and found the accuracy, precision, sensitivity, and specificity of it were 0.928, 0.919, 0.944, and 0.910, respectively (Figure 4c). Then we conducted receiver operating characteristic (ROC) analysis with 2000 stratified bootstrap replicates (Figure 4d). The area under the ROC curve (AUC) value was 0.927 (*95% confidence interval* [*CI*] = 0.884–0.971).

By drawing the ROC curve and using Youden’s index (sensitivity + specificity −1), we found out the optimum critical value. When Youden’s index reaches the maximum, the corresponding value is the optimum critical value. The Y cutoff point of the equation for patients with fungal infection was not less than 1.5.

## Discussion

By establishing a model of fungal infection after transplant in mice, the peripheral blood was collected and screened by microarray. After homology analysis and qRT-PCR verification, we found that compared with the control group, the levels of miR-215 and miR-let-7 c increased significantly in plasma of patients with a fungal infection, while the levels of miR-154, miR-193a, NR_036506.1, and NR_027669.1 decreased markedly in their PBMC. Moreover, we further study on the spatial specificity of these biomarkers and found out that, miR-215, miR-let-7 c, and NR_027669.1 were sensitive to fungal infection in all parts of the body, while miR-154 and miR-193a were relatively specific in blood and lung infections. In addition, NR_036506.1 was significantly down-regulated only in lung and abdominal cavity infections. Furthermore, with the assistance of clustering methods, we established a 6-noncoding-RNA detection panel was set up for fungal infection after organ transplantation, and its diagnostic specificity and sensitivity were quite high.

Traditional diagnostic methods for fungal infections have many shortcomings. For instance, the results of the galactomannan (GM) test and fungal glucan (G) test often turn out to be false-positive, because of their cross-reaction with other bacterial components and even semisynthetic penicillin [21,22]. Fungus culture is the gold standard for diagnosing fungal infection, but its course takes a relatively long time that limits its value in the early diagnosis. It takes 24–48 h for *Candida* and *Cryptococcus* and 48–72 h for *Aspergillus* and *Rhizopus*. Though the microscopy test is quick and convenient, it has many sampling restrictions and a low detection rate, especially for the diagnosis of deep fungal infections. The analysis of fungal metabolites in serum by mass spectrometry is a novel method, but its testing equipment is too expensive. A series of molecular diagnostic methods are developed based on a polymerase chain reaction. It works mainly through the amplification and detection of fungal conservative sequences or specific gene fragments. However, its specificity varies with primer design and sample purity.

MiRNA and lncRNA play an important role in numerous life activity including epigenetic inheritance, cell cycle, and regulation of cell differentiation [23–26]. They change rapidly during the occurrence and development of diseases, and sensitively reflect the dynamic changes of the microenvironment. Herein, compared with the above methods, our 6-noncoding-RNA detection panel has the following advantages. Firstly, the expression of the detected index is stable, not affected by drugs like immunosuppressants. Moreover, ncRNA is a reactive product against pathogen invasion, which can be detected in the early stage of infection, especially when the infection is comparably limited. In addition, only a small amount of peripheral blood samples is needed for rapid and accurate diagnosis, which is convenient for clinical application.

The previous study has indicated miR-215 is up-regulated in pulmonary epithelial cells when infected by *Pseudomonas aeruginosa*, thus activating the NF-κB signaling pathway and inducing inflammation [27]. While in the model of oviduct inflammation, Ibrahim S et al see a decline of miR-155 and miR-215 in oviductal cells after lipopolysaccharide treatment for 24 hours. Meanwhile, proinflammatory mediator tumor necrosis factor-α and interleukin-1β are up-regulated significantly in cell culture [28]. It suggests that, when stimulated by infection, oviductal cells may release miR-215, which acts on inflammatory cells and immune cells through the circulatory system and thus plays a role in the recruitment, chemotaxis, and activation of immune cells. Our research finding that miR-215 increases during fungal infection are consistent with the above reports.

Similarly, miR-let-7 c is significantly increased in the pulmonary epithelial cells of patients with the IV influenza virus. miR-let-7 c binds to the 3’-UTR on the viral gene M1 (+) cRNA to down-regulate the expression of M1, thereby inhibiting the replication of the IV influenza virus [29]. It means that miR-let-7 c can protect host cells from pathogen attacks. Several reports have identified the role of miR-let-7 c in the regulation of immune cells’ development. By binding to the untranslated regions of B lymphocyte induced maturation protein 1 and interferon regulatory factor 4, miR-let-7 c suppresses the expression of these proteins, thus inhibiting the over-differentiation of B lymphocytes into plasma cells and promoting the diversity of B lymphocytes differentiation in the germinal center [30]. That may be the reason why miR-let-7 c increases in patients with fungal infection in our study.

The expression of miR-193a is correlated negatively with PepT1 in the inflamed colon tissue of ulcerative colitis. miR-193a inhibits the NF-κB pathway by reducing the expression and activity of PepT1, thereby maintaining intestinal balance [31]. During the infection, miR-193a will be down-regulated, thereby up-regulating the expression of PepT1, promoting inflammation occurs. This phenomenon is consistent with the downregulation of miR-193a in PBMC of patients with a fungal infection in our research. Moreover, proinflammatory cytokines, like IL-12, are up-regulated in patients with post-traumatic stress disorder, and miRNA microarray shows that miR-193a suppresses the expression of IL-12 [32]. It suggests that the downregulation of miR-193a can promote the expression of proinflammatory cytokines, thereby activating and recruiting inflammatory cells.

The present research on miR-154 mainly focuses on cancer. It is reported that miR-154 inhibits the growth and metastasis of stomach, bladder, and breast cancer [33–35]. The expression of miR-154, miR-376b, and miR-431 decrease in PBMC from patients with initial Graves’ disease (GD), and their expression levels are restored when GD is in remission [36]. It indicates that the downregulation of miR-154 is associated with the occurrence and development of individual immune responses. We also find an apparent decline of miR-154 in PBMC from patients with a fungal infection after organ transplant, which may be related to the immune activation and improvement. But the role and mechanism of miR-154 in the immune response, especially in the battle against pathogenic microorganisms, are still unclear.

Meanwhile, we also discover two lncRNAs that can assist the diagnosis of infectious complications after organ transplantation, including NR_036506.1 (PPIAP30, Peptidylprolyl Isomerase A pseudogene 30), and NR_027669.1 (RNF170, ring finger protein 170). However, so far, due to the lack of relevant research on their exact functions, we are still unclear about their role in the process of fungal infection.

The current research has some limitations. Firstly, we cannot know the exact time when the patient is infected with the pathogen. We can only obtain specimens and information through follow-up of patients with fever. Therefore, there is temporal heterogeneity between specimens. At the same time, our monitoring cases are relatively lacking for the dynamic changes of biomarkers during the infection process. Moreover, we only use computer modeling to verify the specificity and accuracy of diagnostic methods, which means that multi-center and large-sample clinical trials are still needed. Additionally, our model cannot distinguish between specific infected fungal species, such as molds and yeasts, which should be modified in future studies. Finally, the specific mechanisms of related miRNA and lncRNA in the infection process have not been fully elucidated.

## Conclusion

We found that compared with normal transplanted patients, miR-215 and miR-let-7 c were up-regulated in the plasma of patients with a fungal infection, while levels of miR-154, miR-193a, NR_027669.1, and NR_036506.1 were down-regulated in their PBMC. Moreover, by the clustering method, a 6-noncoding-RNA classification of fungal infections after transplantation was established based on the expression of the abovementioned RNAs. Though this classification has excellent diagnostic efficacy, its clinical value still needs to be deeply confirmed by multi-center prospective clinical trials.

## Supplementary Material

Supplemental Material

## Data Availability

The data of microarray was listed in the supplemental materials. The availability of other data and information on the data set used and analyzed in the current study can be provided by the corresponding author upon request.
