# Supplementary material for: Comprehensive analysis of peripheral blood non-coding RNAs identifies a diagnostic panel for fungal infection after transplantation
Source: Bioengineered. 2022 Feb 6;13(2):4039–50. doi: 10.1080/21655979.2022.2032963 (PMC8974173; doi:10.1080/21655979.2022.2032963)
Supplement: Supplemental Material [file KBIE_A_2032963_SM3031.zip › supplementary/ts1clean.docx]

Table S1. The sequences of reverse transcription primers for miRNAs were used in this study.

| Target | RT primer |
| --- | --- |
| miR-215 | CCTGTTGTCTCCAGCCACAAAAGAGCACAATATTTCAGGAGACAACAGGGTCTGTC |
| miR-let-7c | CCTGTTGTCTCCAGCCACAAAAGAGCACAATATTTCAGGAGACAACAGGAACCATA |
| miR-154 | CCTGTTGTCTCCAGCCACAAAAGAGCACAATATTTCAGGAGACAACAGGCGAAGGC |
| miR-193a | CCTGTTGTCTCCAGCCACAAAAGAGCACAATATTTCAGGAGACAACAGGTCATCTC |
