# Supplementary material for: Comprehensive analysis of peripheral blood non-coding RNAs identifies a diagnostic panel for fungal infection after transplantation
Source: Bioengineered. 2022 Feb 6;13(2):4039–50. doi: 10.1080/21655979.2022.2032963 (PMC8974173; doi:10.1080/21655979.2022.2032963)
Supplement: Supplemental Material [file KBIE_A_2032963_SM3031.zip › supplementary/ts2.docx]

Table S2. The sequences of qPCR primers were used in this study.

| Target | Forward primer | Reverse primer |
| --- | --- | --- |
| miR-215 | CGGGCATGACCTATGAATT | CAGCCACAAAAGAGCACAAT |
| miR-let-7c | CGGGCTGAGGTAGTAGGTTG | CAGCCACAAAAGAGCACAAT |
| miR-154 | CGGGCTAGGTTATCCGTGTT | CAGCCACAAAAGAGCACAAT |
| miR-193a | CGGGCTGGGTCTTTGCGGGC | CAGCCACAAAAGAGCACAAT |
| NR_036506.1 | GCGCTATGGGTCCAAGAATGG | CAAATGGGGTGGAGGGTGTTC |
| NR_027669.1 | TGTGGCAGTTGTGGTCAGTT | GCTCCTGGTTTTCTGGGTGA |
| GAPDH | GGACCTGACCTGCCGTCTAG | GTAGCCCAGGATGCCCTTGA |
| U6 | CTCGCTTCGGCAGCACA | AACGCTTCACGAATTTGCGT |
