# Supplementary material for: Comprehensive analysis of peripheral blood non-coding RNAs identifies a diagnostic panel for fungal infection after transplantation
Source: Bioengineered. 2022 Feb 6;13(2):4039–50. doi: 10.1080/21655979.2022.2032963 (PMC8974173; doi:10.1080/21655979.2022.2032963)
Supplement: Supplemental Material [file KBIE_A_2032963_SM3031.zip › supplementary/ts3clean.docx]

|  | Control group  (n=72)† | Fungus-infected group  (n=67)† |
| --- | --- | --- |
| Gender |  |  |
| Female | 7 (9.72%) | 8 (11.94%) |
| Male | 65 (90.28%) | 59 (88.06%) |
| Age (years) | 47 (13-70) | 51 (10-72) |
| Diabetes Mellitus | 10 (13.89%) | 14 (20.90%) |
| HBV Infection before Transplantation | 33 (45.83%) | 29 (43.28%) |
| HCV Infection before Transplantation | 2 (2.78%) | 2 (2.99%) |
| Type of Transplantation |  |  |
| Kidney | 11 (15.27%) | 25 (37.32%) |
| Liver | 58 (80.56%) | 41 (61.19%) |
| Heart | 1 (1.39%) | 0 (0.00%) |
| Hepatopancreaticoduodenal Cluster | 2 (2.78%) | 1 (1.49%) |
| Interval since Transplantation (months) | 11 (1-100) | 1 (1-180) |
| Kinds of immunosuppressive agents | 2 (1-3) | 2 (1-3) |
| With Tacrolimus | 43 (59.72%) | 33 (49.25%) |
| With Mycophenolate Mofetil | 30 (41.67%) | 25 (37.31%) |
| Types of Postoperative Infection |  |  |
| *Candida* | - | 43 (64.18%) |
| *Aspergillus* | - | 11 (16.42%) |
| *Rhizopus* | - | 7 (10.45%) |
| *Cryptococcus* | - | 6 (8.95%) |

Table S3. Demographic and clinical characteristics of the two groups of patients.

†median (range) or number (percentage); HBV, hepatitis B virus; HCV, hepatitis C virus.
