# Supplementary material for: Comprehensive analysis of peripheral blood non-coding RNAs identifies a diagnostic panel for fungal infection after transplantation
Source: Bioengineered. 2022 Feb 6;13(2):4039–50. doi: 10.1080/21655979.2022.2032963 (PMC8974173; doi:10.1080/21655979.2022.2032963)
Supplement: Supplemental Material [file KBIE_A_2032963_SM3031.zip › supplementary/ts4.docx]

Table S4. The importance of components in the Principal Component Analysis (PCA).

| Components | PC1 | PC2 | PC3 | PC4 | PC5 | PC6 |
| --- | --- | --- | --- | --- | --- | --- |
| Standard deviation | 1.5114 | 1.2163 | 1.0862 | 0.72652 | 0.59944 | 0.4113 |
| Proportion of variance | 0.3807 | 0.2466 | 0.1966 | 0.08797 | 0.05989 | 0.0282 |
| Cumulative proportion | 0.3807 | 0.6273 | 0.8239 | 0.91191 | 0.97180 | 1.0000 |
